# Neutrophils suppress adaptive αβ T cells but not the innate γδ T cells and natural killer cells

**DOI:** 10.3389/fimmu.2026.1977867

**Published:** 2026-10-01

**Authors:** Angela A.F. Gankema, Fleur van Oosterom, Manon C. Slot, Céline I. Mahieu, Stamatia Rontogianni, Tamara Verkerk, Derk Amsen, Robin van Bruggen, Taco W. Kuijpers

**Affiliations:** 1 Sanquin Research, University of Amsterdam, Amsterdam, Netherlands; 2 Department of Pediatric Immunology, Rheumatology and Infectious Diseases, Emma Children’s Hospital, Amsterdam University Medical Center (Amsterdam UMC), University of Amsterdam, Amsterdam, Netherlands

**Keywords:** immunotherapy, MDSC activity, neutrophils, NK cells, T cell subsets

## Abstract

The presence of polymorphonuclear neutrophils (PMNs) in the tumor microenvironment is associated with lower survival rates for cancer patients. Presumably due to the immunosuppressive activity of neutrophils (also known as Myeloid-Derived Suppressor Cell (MDSC) activity). We investigated whether this MDSC activity shows any selectivity in suppressing specific lymphocyte subsets. Our data show that CD4^+^ and CD8^+^ T cells, both naive and memory fractions, are sensitive to PMN-MDSC activity. Similarly, regulatory T cells, believed to play an important immunosuppressive function themselves, were affected too. In contrast, the more innate-like γδ T cells and Natural Killer (NK) cells were left intact. Our data suggest that Chimeric Antigen Receptor (CAR)-NK and γδ CAR-T cell immunotherapy may be less susceptible to PMN-MDSC activity. Therefore, using such MDSC-resistant innate cells as cellular products may be of advantage over the common αβ CAR-T cell therapies. Additionally, further deciphering the exact mechanisms by which neutrophils suppress αβ T cells could lead to improved T cell immunotherapies.

## Introduction

Cancer is the second leading cause of mortality in the western world (1). In recent years there have been substantial improvements in cancer immunotherapy, including tumor-infiltrating lymphocyte (TIL) and Chimeric Antigen Receptor (CAR)-T cell therapy (2–4). There are seven FDA-approved CAR-T cell therapies on the market targeting CD19 for lymphomas and leukemia or targeting B Cell Maturation Antigen (BCMA) for multiple myeloma (5). These CAR-T cell therapies show dramatic clinical responses in hematologic malignancies (2). Nevertheless, therapeutic effects in solid tumors are not durable as they are known to create an immunosuppressive environment that supports tumor growth (6). Emerging evidence suggests that the effectiveness of immunotherapy can be maximized by also addressing this immunosuppressive tumor microenvironment (TME) (7–10).

Containing a variety of different immune cells as well as a myriad of local soluble factors, the TME is immunologically highly diverse and inherently complex (11–13). While tumor infiltration of some immune cell types such as CD8^+^ T cells, Natural Killer (NK) cells, and γδ T cells are described to be favorable for patient outcome, other immune cells like monocytes and neutrophils are disadvantageous for patient outcome (8, 14). A high neutrophil-to-lymphocyte ratio in blood as well as a high number of immunosuppressive Regulatory T cells (Tregs) in the TME is also associated with a general poor prognosis in cancer patients (15–17).

Polymorphonuclear neutrophils (PMNs) are part of the innate immune response and are able to clear pathogens using a variety of mechanisms. These include cytokine production, release of reactive oxygen species (ROS), degranulation to release antimicrobial peptides, proteins and enzymes, NETosis and phagocytosis (18). However, in a tumor setting, neutrophils can also suppress T cells (19). This is a process called myeloid-derived suppressor cell (MDSC) activity which results in immunosuppressed, damaged T cells which can no longer proliferate and are unable to exert their anti-tumor function (19).

In recent years, a few studies have been conducted into neutrophil-mediated MDSC activity towards CD3^+^ T cells (19). The two main conventional T cell subsets tested in the context of cocultures with activated neutrophils, are the CD4^+^ T helper cells and CD8^+^ cytotoxic T cells, known for their tumor killing potential (19, 20). However, the effect of PMN-MDSC activity on other adaptive immune subsets such as Tregs, or innate immune cells such as γδ and NK cells, is largely unknown. Limited studies are performed on γδ T cells, and the studies that are available show inconsistent results, indicating either an inhibitory effect of neutrophils on γδ T cell proliferation or no effect (21, 22). While one study has shown that neutrophils from healthy donors receiving granulocyte colony-stimulating factor (G-CSF) for stem cell mobilization could inhibit NK cell-mediated tumor cell cytotoxicity, not much else is known about the suppressive effect of neutrophils on NK cell proliferation itself (23). Another study showed an inverse correlation between the number of neutrophils present in blood and tumor lesions of cancer patients, and the number of NK cells found (12). Based on these results it has been suggested that neutrophils could suppress NK cell infiltration, proliferation or functionality (12). Neutrophils are known to perform MDSC activity towards T cells but whether this suppression extends towards different subsets of T cells or NK cells has not been comprehensively studied. Knowing whether this PMN-MDSC activity is selective for certain subsets would be highly relevant to further dissect the mechanisms of PMN-MDSC activity. In addition, this knowledge can help to advance current immunotherapies like TIL, CAR-T and future CAR-NK cell therapies by engineering these cells in such a way that they are equipped to resist the immunosuppressive activity of neutrophils, improving therapy success rates. To investigate whether PMN-MDSC activity shows any selectivity towards specific T lymphocyte subsets, and extends towards innate immune cells, we used a well-characterized *in vitro* assay to assess human neutrophil immunosuppressive activity towards different αβ T cell subsets, innate-like γδ T cells and NK cells. Our study demonstrates that PMN-MDSC activity is restricted to αβ-TCR bearing T cell subsets, but does not affect innate γδ T cells or NK cells.

## Materials and methods

### Blood cell isolation

In accordance with Dutch regulations following the Declaration of Helsinki, blood from anonymized, healthy adults who have given informed consent approved by Sanquin internal Ethical Advisory Board (Privacy Declaration 1909-0176), was collected in heparin tubes. In total blood from 40 different donors was used. These donors showed an even sex distribution and age between 18–66 years. This fresh blood was diluted with phosphate-buffered saline (PBS) containing 10% (v/v) trisodium citrate (TNC) in a 1:1 ratio. Blood was layered on isotonic Percoll (Pharmacia, 1.076 g/mL) and immune cells were separated by density centrifugation for 20 minutes at 800g at room temperature (RT). The peripheral blood mononuclear cells (PBMCs) were collected, washed and used to isolate T cells or NK cells by magnetic-activated cell sorting (MACS). To isolate T cell subsets and NK cells, pan T cell isolation kit, CD4^+^ T cell isolation kit, CD8^+^ T cell isolation kit, naive CD4^+^ T cell isolation kit, memory CD4^+^ T cell isolation kit, naive CD8^+^ T cell isolation kit, memory CD8^+^ T cell isolation kit, γδ T cell isolation kit, and NK cell isolation kit from Miltenyi-Biotec were used according to the manufacturers protocol. The isolation efficiency of the cells was assessed (Supplementary data) and cells with a purity over 80% were used in functional assays. To purify neutrophils from the pellet fraction remaining after density centrifugation, ice-cold erythrocyte lysis buffer (150 mM NH_4_Cl, 10 mM KHCO_3_, and 0.1 mM EDTA) was used to lyse red blood cells at 4°C. Neutrophils were washed and resuspended in complete IMDM (IMDM (Gibco Life Technologies) supplemented with fetal calf serum (FCS) (10% v/v, Bodinco), L-glutamine (final concentration of 200mM, Sigma Aldrich), penicillin (final concentration of 10^4^ U/mL, Sigma Aldrich) and streptomycin (final concentration of 10 ng/mL, Sigma Aldrich)) at 1.2 10^6^ cells/mL.

### Treg culture

CD4^+^ T cells were isolated from the PBMC fraction and subsequently sorted by FACS for CD25^high^CD127^dim^ cells and GPA33^+^ cells (stabile Tregs) (24, 25). Tregs were expanded for one week in complete IMDM supplemented with IL-2 (300 U/mL, Proleukin), anti-CD3 (0.1 µg/mL, Clone 1XE [IgE isotype], Sanquin) and anti-CD28 monoclonal antibody (0.1 µg/mL, CD28.2, eBioscience, RRID: AB_467194) at 37 °C. A medium refreshment was performed halfway through the cell culture period. After one week in culture, the cells were harvested and a fraction was permeabilized, stained for intracellular anti-FOXP3-PECy7 (Clone 236A/E7, eBioscience, RRID: AB_2573450) and anti-Helios-PerCPCy5.5 (Clone 22F6, Biolegend, RRID: AB_2561640) staining as additional validation of Treg purity. Eventually, Tregs with a purity of >80% were used in T cell proliferation assays.

### NK cell culture

After NK cell isolation, cells were kept for one week in NK MACS medium (Miltenyi) (supplemented with 1% NK MACS supplements, 5% human serum, 5% FCS, 1% L-glutamine (Sigma Aldrich), 1% penicillin and streptomycin (Sigma Aldrich), IL-2 (10 ng/mL) and IL-15 (10 ng/mL, Peprotech) at 37 °C. Medium was refreshed every two to three days. After being in culture for one week, cells were harvested and the purity of cells was assessed with a staining of anti-CD56-PerCPCy5.5 (clone HCD56, 1:50, BioLegend, RRID: AB_893389) and anti-CD3-PB (clone UCHT1, 1:500, Invitrogen, RRID: AB_2536467). Cells with a purity of at least 80% were counted and used in a coculture with neutrophils.

### Tumor cell culture

The human erythroleukemia cell line K562 (ATCC) expressing a turquoise-LMNB1 + Her2Neu construct was routinely cultured in IMDM supplemented with 20% FCS, L-glutamine (200 mM), penicillin (10^4^ U/mL) and streptomycin (10 ng/mL) at 37 °C and 5% CO_2_.

### T/NK cell proliferation assay

Purified T or NK cells were labeled with carboxyfluorescein succinimidyl ester (CFSE) proliferation dye (0.5 µM, Molecular Probes) for eight minutes in a shaking water bath at 37 °C. Cells were washed twice with PBS and resuspended in complete IMDM at 0.4 10^6^ cells/mL. T or NK cells were cocultured with neutrophils in a 1:3 ratio (20, 000 T or NK cells and 60, 000 neutrophils) in 96-well flat bottom plates at 37 °C. αβ T cells were stimulated with anti-CD3 (Clone 1XE [IgE isotype], Sanquin) and anti-CD28 (Clone 15E8 [IgG1 isotype], Sanquin) monoclonal antibodies in suspension (5 µg/mL). γδ T cells were stimulated with IL-2 (120 U/mL, Peprotech), IL-7 (20 ng/mL, Research grade, Miltenyi), IL-15 (20 ng/mL), and phytohaemagglutinin (PHA) (1 µg/mL, HA16 Remel™, Thermo Fisher Scientific). NK cells were stimulated with IL-2 and IL-15 (10 ng/mL). Neutrophils were stimulated with TNFα (10 ng/mL, Peprotech EC, London, United Kingdom). After four days of coculture, T or NK cell proliferation was assessed based on CFSE intensity by flow cytometry. The formation of damaged cells was determined by forward/side scatter. To determine cell purity, T cells were labelled with anti-CD4-APC (clone SK3, 1:200, BD Biosciences, RRID: AB_2739445), anti-CD8-PerCP-Cy5.5 (clone SK1, 1:500, BioLegend, RRID: AB_2044009), anti-CD45RA-PECy7 (clone HI100, 1:200, BD Biosciences, RRID: AB_1727498), anti-CD45RO-PB (clone UCHL1, 1:200, BioLegend, RRID: AB_493659), anti-CD27-FITC (clone O323, 1:100, BioLegend, RRID: AB_314298) or anti-γδTCR-PE (clone REA591, 1:100, Miltenyi Biotec, RRID: AB_2751121). NK cells were identified with anti-CD56-PerCPCy5.5 (clone HCD56, 1:50, BioLegend, RRID: AB_893389). **Supplementary Table 1** gives an overview of the source, purity, expansion history, stimulation condition, neutrophil treatment and biological replicate number for the different isolated lymphocyte populations.

### NK cell cytotoxicity

NK cells were cocultured with neutrophils in presence of IL-15 and TNFα. After 1–2 days, neutrophils will die and therefore only viable NK cells will be left. On day four of the coculture, NK cells were harvested, counted and cocultured with the human erythroleukemia cell line K562 (RRID: CVCL_0004) turquoise-LMNB1tag + Her2neu cells in presence of IL-15 in a NK:K562 ratio of 5:1. To control for spontaneous tumor cell death, K562 cells were cultured solely and for maximum tumor cell killing, 0.1% triton was added for 30 minutes at 37 °C. After four hours, tumor cell killing by NK cells was assessed via flow cytometry using live/dead near IR (Thermo Fisher Scientific). Subsequently, the turquoise tag expressed by the K562 cells was used to gate the tumor cells, followed by a gate for single cells and the percentage of dead cells.

### Trogocytosis

T cells were labelled either with the lipid-intercalating dye DiD or DiO (1:1200, ThermoFisher Scientific) and neutrophils were labelled with Celltrace calcein red-orange (1:10, 000, ThermoFisher Scientific) for 30 minutes in a shaking waterbath at 37 °C (26). Cells were washed with PBS twice and resuspended in complete IMDM. Both T cells and neutrophils were added in a 1:2 ratio to 8-well Ibidi chambers (Ibidi). Anti-CD3/anti-CD28 monoclonal antibodies (both at 5 µg/mL) and TNFα (10 ng/mL) were added in suspension. Cells were imaged with the SP8 (Leica) confocal microscope for four hours at 37 °C. Videos were recorded with a time frame of 1 picture/56 seconds. Data processing was performed Fiji (Fiji, Schindelin et al., RRID: SCR_002285) For Imagestream analysis, T cells were cocultured with neutrophils in a 1:3 ratio for 180 minutes at 37 °C, whereafter fixed (20 mM NAF, 0.5% PFA, and 1% BSA in PBS) and measured by ImageStream Mark II (Merck). Data were analyzed with software program IDEAS^®^ (Amnis Corporation).

### Statistical analysis

Statistical tests were performed using Graphpad Prism 10 (RRID: SCR_002798). Normality was tested using the Shapiro-Wilk normality test. For sample sizes of n=3-4, it was assumed that the data were normally distributed. Data were further analyzed using either an RM one-way ANOVA followed by Dunnett’s *post hoc* multiple-comparison test or a Friedman one-way ANOVA followed by Dunn’s *post hoc* multiple-comparison test. Sample groups were compared to the control group without neutrophils and TNFα. For the CD4naive, CD4memory and Treg proliferation data, internal comparisons were performed by using RM one-way ANOVA corrected for multiple comparisons by controlling the False Discovery Rate (original FDR method of Benjamini and Hochberg). Significance was assessed using adjusted q-values. Data are represented as the median and interquartile range. All datapoints show results from independent donors. P values <0.05 were considered statistically significant. The specific tests used per experiment can be found in the figure legends.

## Results

### CD4^+^ and CD8^+^ T cell proliferation is suppressed by PMN-MDSC activity

To study if both CD4^+^ and CD8^+^ T cells are vulnerable towards PMN-MDSC activity, we cultured CD3^+^ T cells from healthy donors in the absence or presence of (TNFα-stimulated) neutrophils and assessed proliferation of different T cell subsets after 4 days. These T cells were cultured with soluble aCD3/aCD28 monoclonal antibodies to induce proliferation. From the single CD4^+^ or CD8^+^ T cells, both T cell proliferation as well as damaged T cell formation were quantified (**Figure 1A**). Irrespective of the coculture with neutrophils, these T cell subsets showed proliferation upon aCD3/aCD28 T cell stimulation (**Figure 1B**). No immediate effect on proliferation of CD4^+^ or CD8^+^ T cells was observed following the addition of TNFα in the absence of neutrophils. However, activation of PMN-MDSC activity through the addition of TNFα, consistently reduced CD4^+^ and CD8^+^ T cell proliferation, confirming our previous findings (19) (**Figure 1B**). Similar results could be found with N-formylmethionine-leucyl-phenylalanine (fMLP) or lipopolysaccharide (LPS) as neutrophil stimulation (data not shown), while TNFα is the strongest and most consistent stimulus. To ensure that the presence of one cell type did not affect the vulnerability of the other, we repeated these experiments with purely isolated CD4^+^ or CD8^+^ T cells as separate cell fractions (**Supplementary Figure 1A**). Although CD8^+^ T cells proliferated slightly less compared to CD4^+^ T cells in this assay, T cell proliferation of both these isolated T cell fractions was likewise suppressed by the TNFα-induced PMN-MDSC activity (**Figure 1C**). To conclude, both conventional CD4^+^ and CD8^+^ T cells are vulnerable to PMN-MDSC activity.

**Figure 1:**
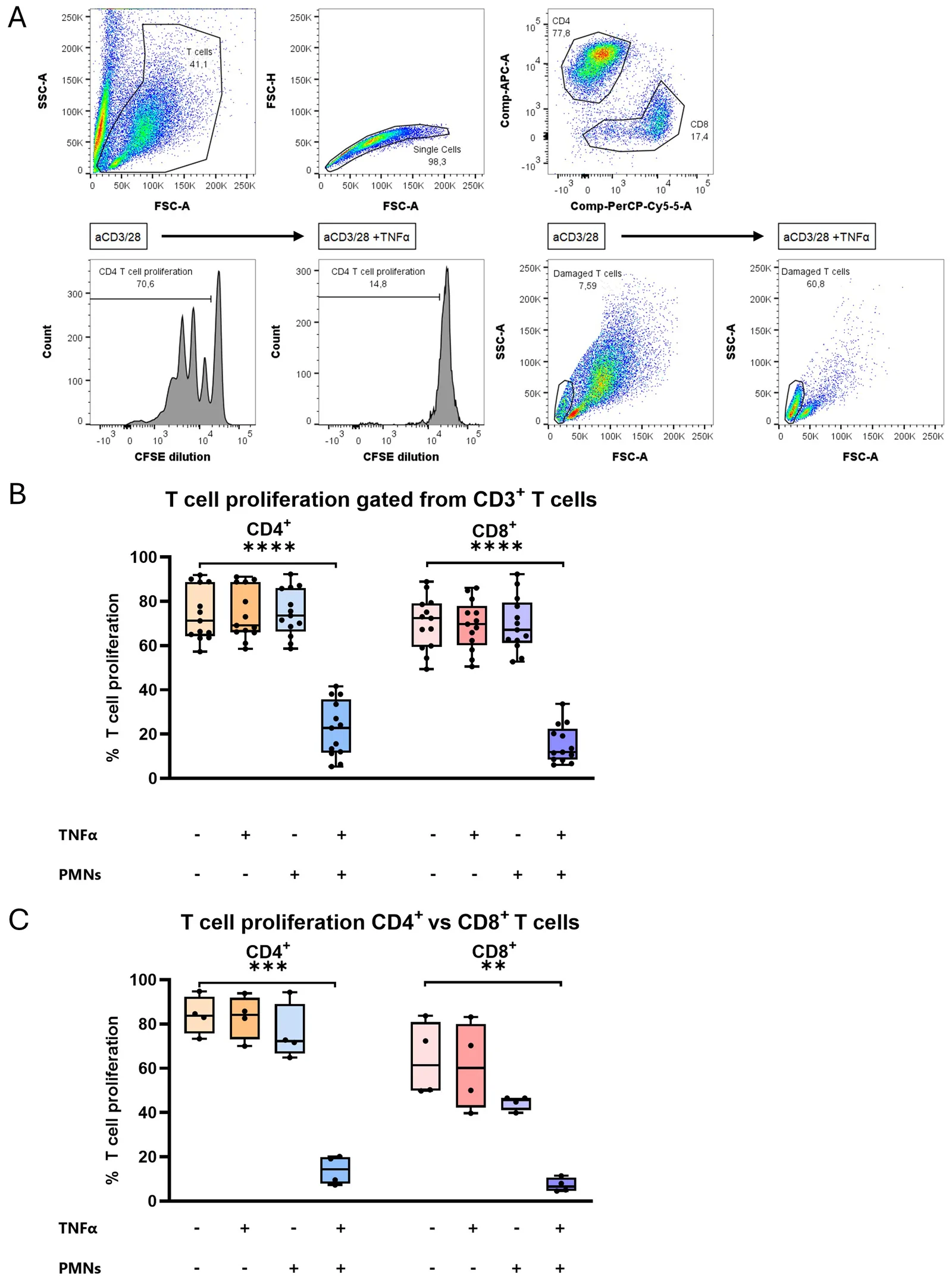
CD4^+^ and CD8^+^ T cells are both suppressed by PMN-MDSC activity. **(A)** Gating strategy used to determine T cell suppression. The first row shows the gates set for T cells, single cells, and CD4^+^ and CD8^+^ T cells. The second row shows how T cell proliferation was determined by CFSE dilution and analysis of damaged T cells by FSC/SSC. As an example, aCD3/28 stimulated T cells are shown in presence of either unstimulated neutrophils or neutrophils stimulated with TNFα. **(B)** CD4^+^ and CD8^+^ T cell proliferation gated on isolated CD3^+^ T cells. n=13. **(C)** Isolated CD4^+^ and CD8^+^ T cell proliferation. n=4. Data are represented as median and interquartile range. P values were calculated using a RM one-way ANOVA followed by Dunnett’s *post hoc* multiple comparison test. **p<0.01, ***p<0.001 ****p<0.0001.

### PMN-MDSC activity affects T cell viability for both CD4^+^ and CD8^+^ T cells

Previous observations showed that PMN-MDSC activity towards T cells, next to inhibition of T cell proliferation, also results in damaged T cells characterized by the decrease in forward/side scatter (19). This damaged T cell population is the result of neutrophil trogocytosis. To visualize cell-cell contact between CD4^+^ or CD8^+^ T cells and neutrophils, we performed confocal microscopy (**Supplementary Video 1**). TNFα-stimulated neutrophils were found to perform trogocytosis when incubated with both CD4^+^ or CD8^+^ T cells. These results were also confirmed with analysis of uptake of labeled T cell membrane parts by neutrophils analyzed with the Imagestream (**Figure 2A**). Next, we assessed whether damaged T cells could be observed for the individual T cell subsets (**Figure 1A**). Both CD4^+^ and CD8^+^ T cells showed damaged T cells on day 4 of the coculture with TNFα-stimulated neutrophils (**Figure 2B**). Similarly, isolation of CD4^+^ and CD8^+^ T cells as separate cell fractions, showed damaged T cells only upon activation of PMN-MDSC activity by TNFα (**Figure 2C**). Tracing back the damaged T cell population (blue) in the total CD4/CD8 plot (red) showed that the damaged T cells were the cells that were lower in CD4 or CD8 expression, as previously confirmed for a series of surface markers (**Figure 2D**) (19). Furthermore, when T cells were cocultured with neutrophils, this damaged T cell population was present from day one onwards, suggesting that neutrophils exert MDSC activity already during the first period of the coculture (**Supplementary Figure 1B**). In summary, CD4^+^ and CD8^+^ T cells are both vulnerable to trogocytosis by neutrophils, leading to damaged, small T cells.

**Figure 2:**
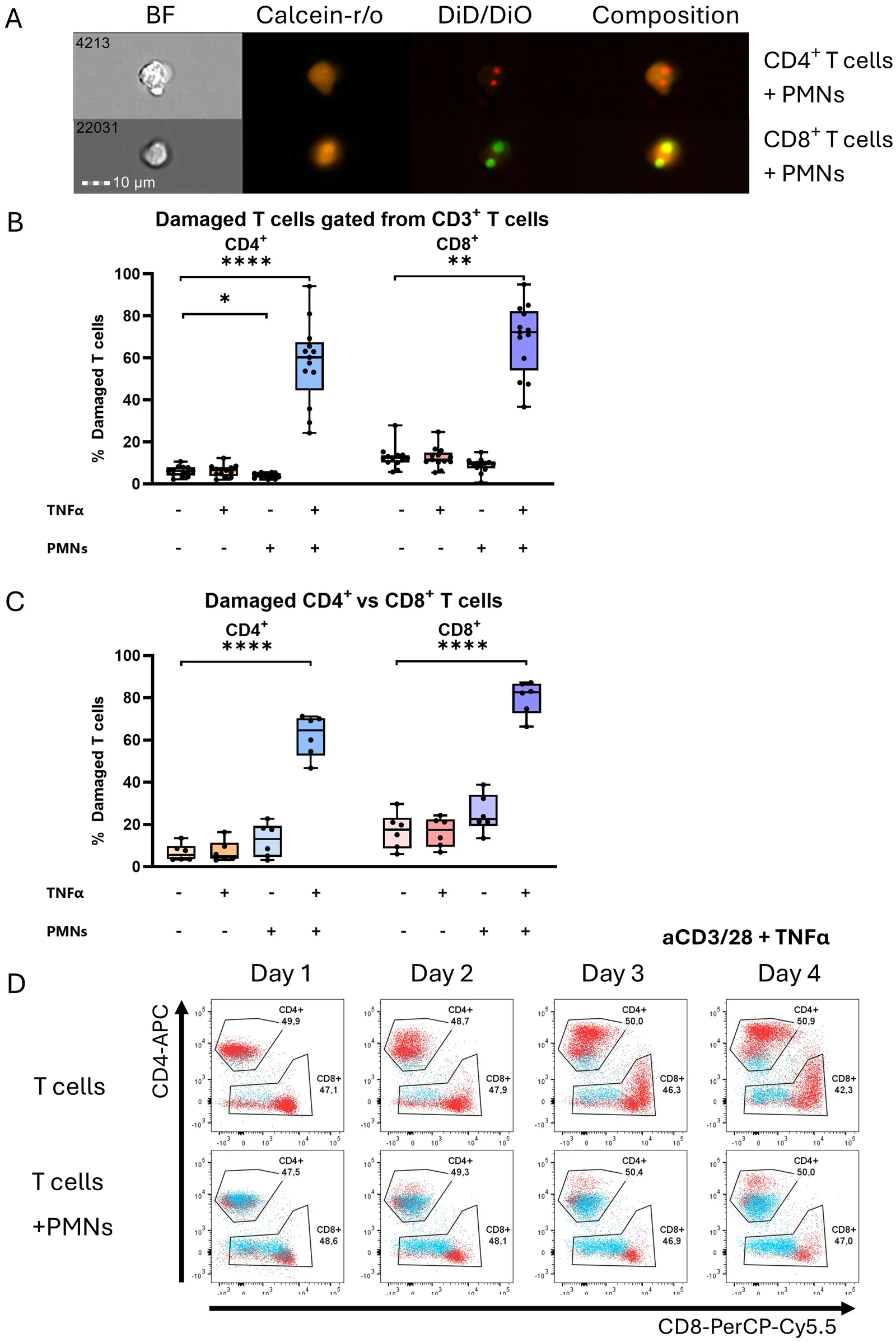
CD4^+^ and CD8^+^ T cells are damaged as a result of PMN-MDSC activity. **(A)** Representative images of trogocytosed T cell membrane parts from CD4^+^ (red) or CD8^+^ (green) T cells by neutrophils (orange) as visualized by imaging flow cytometric analysis. Scale bar was set at 10 µm. T cells were stimulated with aCD3/28 and neutrophils with TNFα. **(B)** Analysis of damaged CD4^+^ and CD8^+^ T cells resulting from a coculture with neutrophils for four days, gated on isolated CD3^+^ T cells. n=13. **(C)** Analysis of damaged CD4^+^ and CD8^+^ T cells resulting from a coculture with neutrophils for four days, gated on isolated CD4^+^ and CD8^+^ T cells. n=6. **(D)** Damaged T cells backgated in the total CD4/CD8 T cell in presence of aCD3/28 and TNFα. Data are represented as median and interquartile range. P values were calculated using a Friedman one-way ANOVA followed by Dunn’s *post hoc* multiple comparison test for **(B)** and a RM one-way ANOVA followed by Dunnett’s *post hoc* multiple comparison test for **(C)** *p<0.05, **p<0.01, ****p<0.0001.

### PMN-MDSC activity is not restricted to the differentiation state of CD4^+^ or CD8^+^ T cells

In the TME, effector memory T cells make up the dominant T cell population (27, 28). Though, it is not known whether this population is relatively more affected by PMN-MDSC activity compared to for example, naive T cells, while knowing would help select for certain markers or interactions in immunotherapy or focus on suppressing PMN-MDSCs in general. Therefore, we wanted to determine whether both naive and memory T cells were vulnerable to PMN-MDSC activity.First, we isolated CD4^+^ naive (CD4^+^CD45RA^+^) and CD4^+^ memory (CD4^+^CD45RO^+^) T cells and determined their vulnerability for PMN-MDSC activity in a separate coculture (**Supplementary Figure 2A**). Proliferation of both CD4^+^ naive and CD4^+^ memory T cells was suppressed by PMN-MDSC activity and damaged T cells were found (**Figures 3A, B**). Next, also CD8^+^ T cells were separated into naive (CD8^+^CD45RA^+^) and memory (CD8^+^CD45RO^+^) T cells and purity of the cells was assessed prior to cell culture (**Supplementary Figures 3A, B**). CD8^+^ naive and CD8^+^ memory T cells were also both suppressed by PMN-MDSC activity and damaged T cells were formed (**Supplementary Figures 4A, B**). Remarkably, in case of both CD4^+^ naïve and CD8^+^ naive T cells, the addition of unstimulated neutrophils already reduced their proliferative capacity, which could be further decreased by TNFα stimulation, whereas CD4^+^ memory and CD8^+^ memory T cells only showed a reduction in proliferation when neutrophils were stimulated with TNFα. To sum up, we found that both naive and memory T cells are affected by PMN-MDSC activity, whereas naive T cells could already be suppressed by unstimulated neutrophils, memory T cells were only suppressed by adding TNFα to stimulate neutrophils.

**Figure 3:**
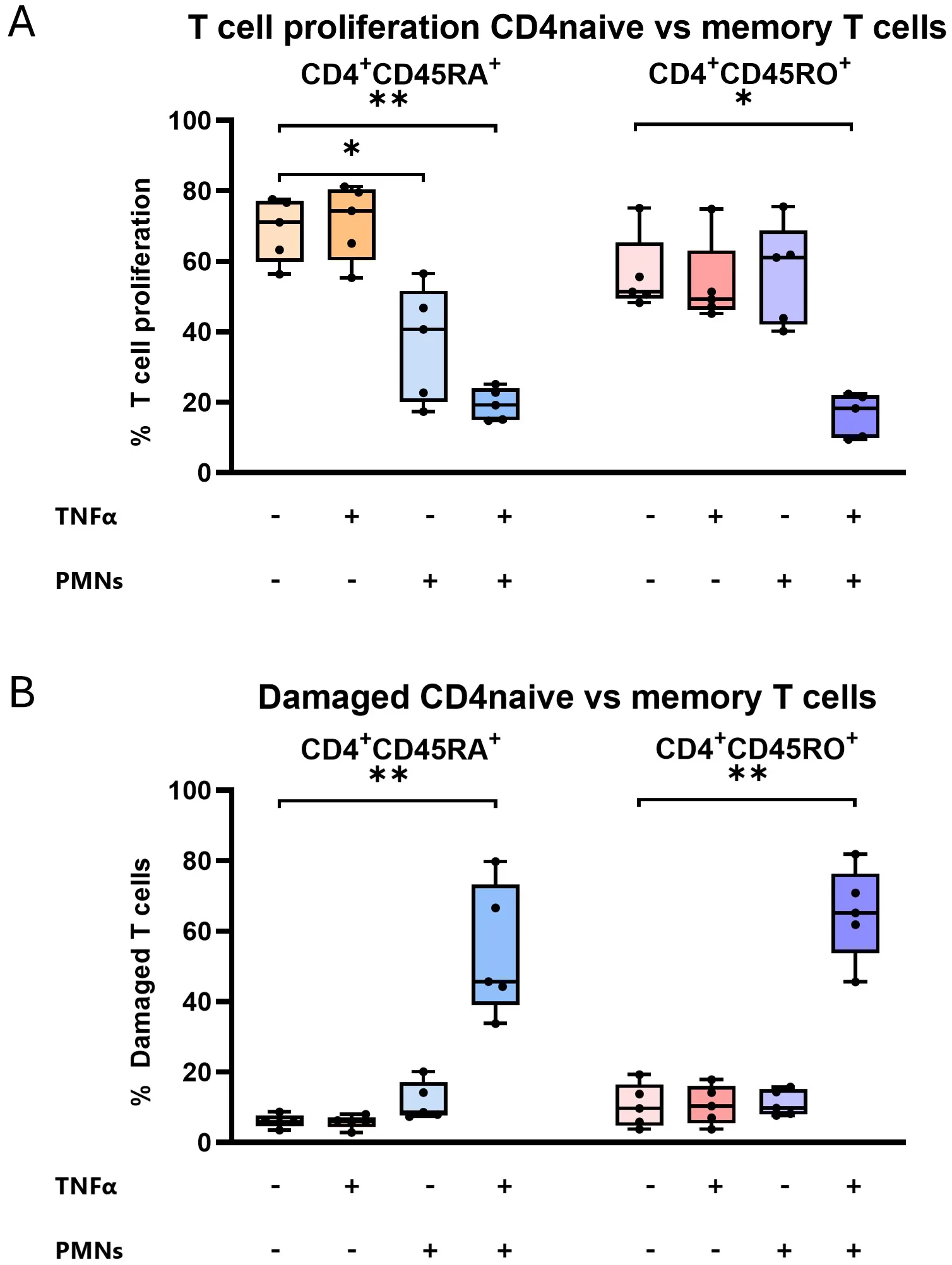
CD4naive and memory T cells are both vulnerable to PMN-MDSC activity. **(A)** CD4naive and CD4memory T cell proliferation. n=5. **(B)** Analysis of CD4naive and CD4memory damaged T cells resulting from a coculture with neutrophils for four days. n=5. P values were calculated using a Friedman one-way ANOVA followed by Dunn’s *post hoc* multiple comparison test for **(A)** and a RM one-way ANOVA followed by Dunnett’s *post hoc* multiple comparison test for **(B)** *p<0.05, **p<0.01.

### Tregs are damaged by PMN-MDSC activity

Tregs are another type of T cells that are also present in the TME (29). However, instead of having anti-tumor activity, these cells have an immunosuppressive function. They play a critical role in immune tolerance and the prevention or mitigation of harmful immune reactions (30–32). MDSC activity is mostly described to be advantageous to tumor proliferation and progression, leading to an immunosuppressive environment too (33). It is, however, unknown if neutrophil- and Treg suppression can occur simultaneously or if neutrophils also suppress Tregs since they are similar in phenotype to the conventional, αβ-TCR+ CD4^+^ T cells tested before. Therefore, we wondered if Tregs are also susceptible to PMN-MDSC activity, which may counteract the suppression of anti-tumor immunity by Tregs as such. To this purpose, CD25^high^CD127^dim^CD4^+^ Tregs were isolated and expanded, after which a T cell proliferation assay was performed (**Supplementary Figure 5A**). Proliferation of Tregs is low without addition of TNFα or neutrophils but did increase when adding either one of them. Adding TNFα-stimulated neutrophils did again decrease T cell proliferation and damaged T cells were formed (**Figure 4A**). These results demonstrate that Tregs are vulnerable to PMN-MDSC activity.

**Figure 4:**
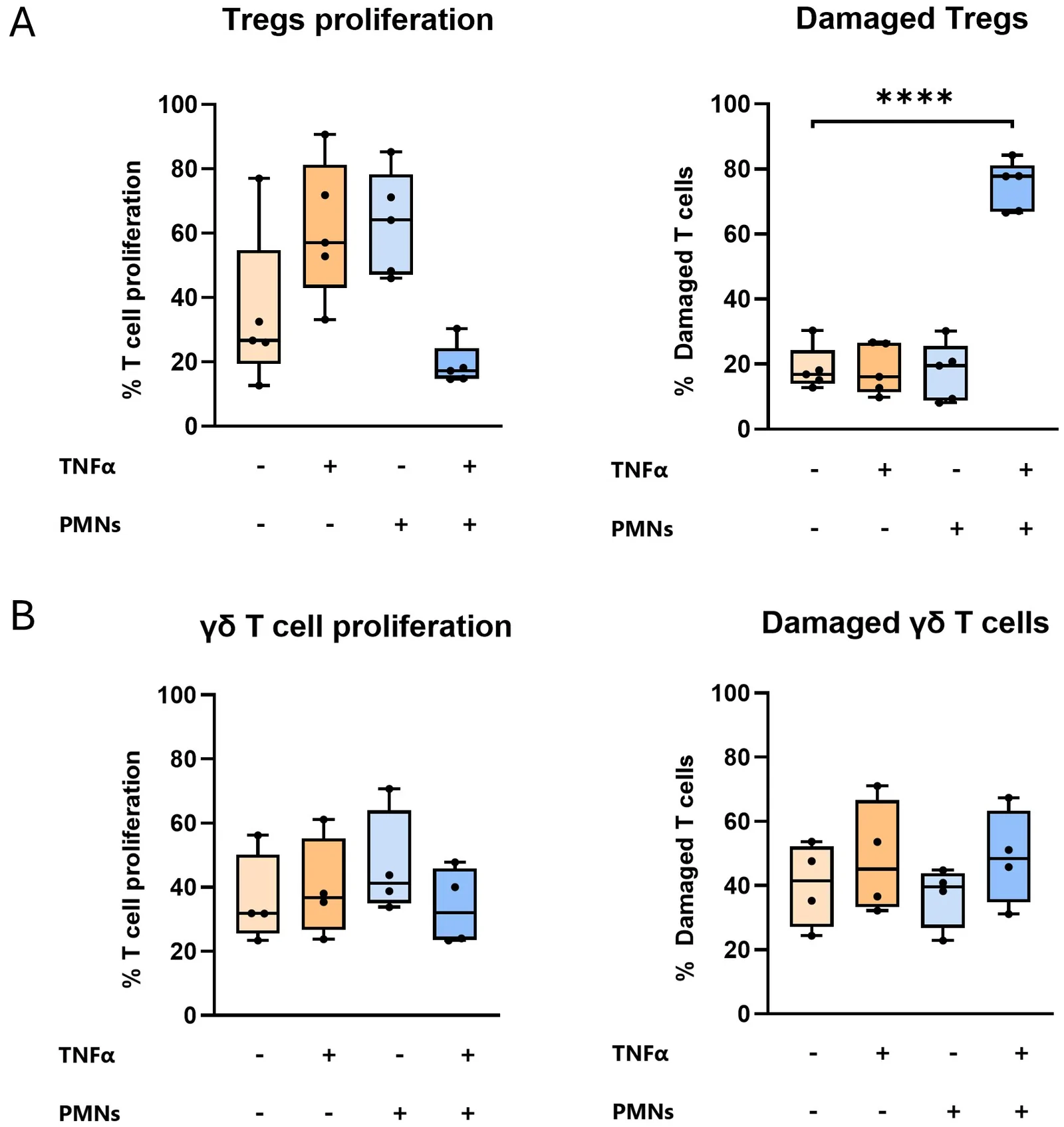
Tregs are vulnerable for PMN-MDSC activity while γδ T cells are not. **(A)** Tregs T cell proliferation and analysis of damaged T cells resulting from a coculture with neutrophils for four days. n=5. **(B)** γδ T cell proliferation and analysis of damaged γδ T cells resulting from a coculture with neutrophils for four days. n=4. P values were calculated using a RM one-way ANOVA followed by Dunnett’s *post hoc* multiple comparison test for damaged Tregs and B and a Friedman one-way ANOVA followed by Dunn’s *post hoc* multiple comparison test for Tregs proliferation. ****p<0.0001.

### PMN-MDSC activity selectively acts on conventional TCR-αβ^+^ T cells and does not affect γδ T cells and NK cells

As of similar abundance as Tregs in the circulation, cytotoxic γδ T cells are also known to infiltrate solid tumors, being part of the TME (34). Because of their MHC-I independent anti-tumor responses, these cells are of high interest for the development of therapies (35, 36). For that reason, and to determine if the suppression observed is restricted to T cells containing the αβ-TCR, we investigated whether these γδ T cells, lacking an αβ-TCR, are vulnerable to PMN-MDSC activity as well.

To assess this, a T cell proliferation assay was performed with isolated γδ T cells and neutrophils (**Supplementary Figure 5B**). In contrast to the conventional αβ-TCR positive T cells, T cell proliferation of the innate γδ T cells was not suppressed by PMN-MDSC activity (**Figure 4B**). In accordance with the unaffected γδ T cell proliferation, no increase in the percentage of damaged γδ T cells was observed when adding TNFα stimulated neutrophils (**Figure 4B**).

NK cells are another innate-like cell type present in the TME with a similar function to γδ T cells (37). NK cells are mainly known for recognizing malignant cells in antigen-independent matters, such as through the absence of MHC-I (37). Since γδ T cells resisted TNFα-induced PMN-MDSC activity, we questioned whether other innate cells found in the TME, such as NK cells, can resist PMN-MDSC activity. To test this, we performed a similar proliferation assay as for the different T cell subsets but this time with NK cells (**Supplementary Figure 5C**). PMN-MDSC activity did not affect NK cell proliferation, nor did it induce damaged NK cells (**Figure 5A**). Additionally, we tested NK cell anti-tumor cytotoxicity after coculture with TNFα-stimulated neutrophils for four days. Using MHC-I negative K562 targets cells expressing a turquoise tag (PB+), we assessed live/dead near IR staining (APC-Cy7+) in a NK cell-mediated killing assay (**Figure 5B**). NK cell cytotoxicity towards K562 target cells was unaltered (**Figure 5C**). NK cells showed high killing capacity of K562 cells, also after a coculture with TNFα-stimulated neutrophils for four days, indicating that TNFα-stimulated PMN-MDSC activity does not affect their ability to kill tumor cells (**Figure 5C**). Thus, PMN-MDSC activity is restricted towards αβ T cells, and does not affect γδ nor NK cells.

**Figure 5:**
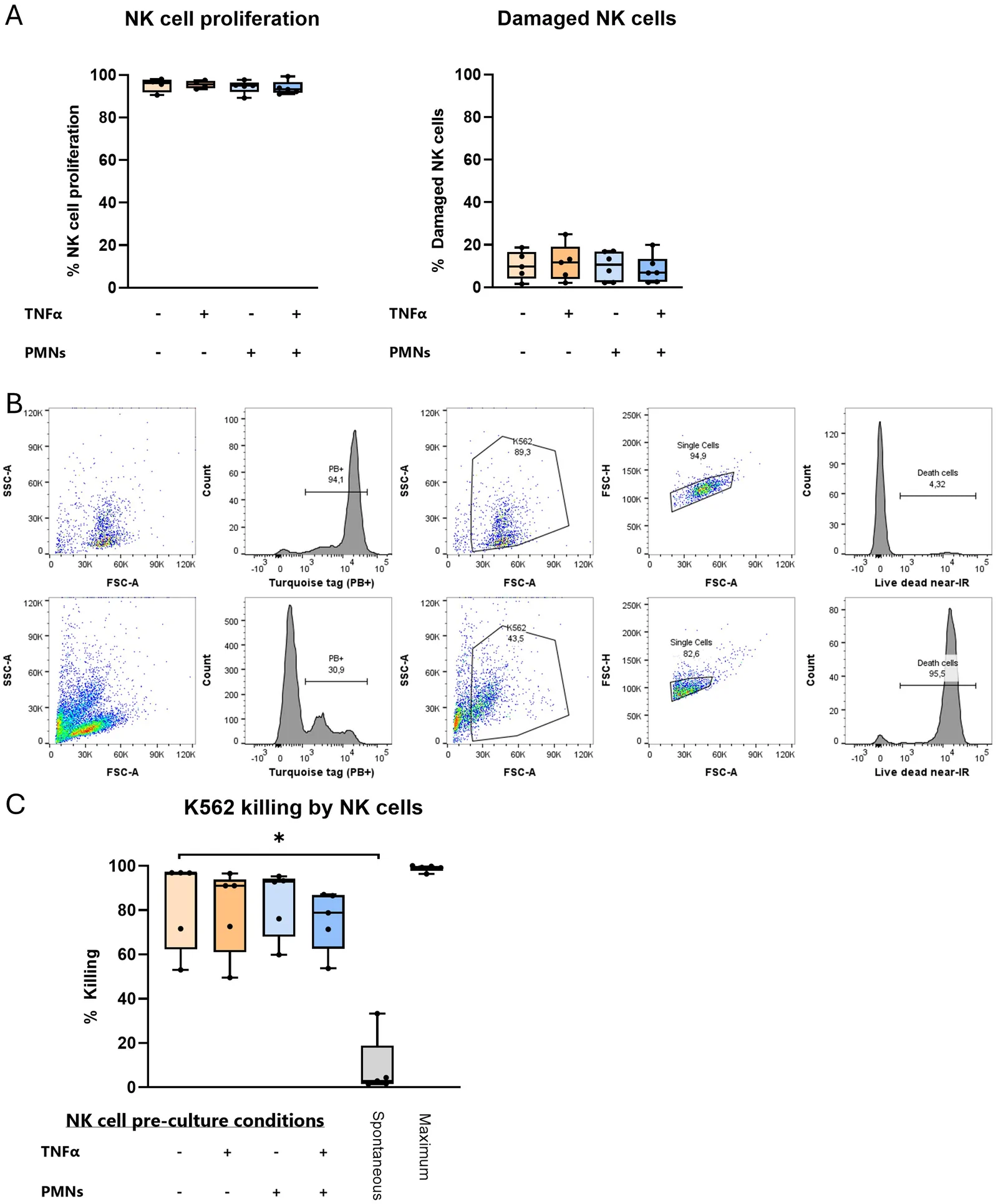
NK cells are not vulnerable to PMN-MDSC activity. **(A)** NK cell proliferation and analysis of damaged NK cells resulting from a coculture with neutrophils for four days. n=5-6. **(B)** Gating strategy used to assess tumor cell killing by flow cytometry. First row is the spontaneous control with tumor cells only and the second row is tumor cells with NK cells + IL-15 stimulation. First, the FSC-A/SSC-A is shown, whereafter we gated for K562 cells by using their turquoise tag (PB+ cells), next we gated out the debris, gated for single cells and gated for dead cells by the live/dead near-IR staining **(C)** K562 (turquoise-LMNB1 + Her2neu) tumor cell death caused by NK cells, which have been in coculture with neutrophils, IL-15 and TNFα for four days prior. Tumor cells only were used as control for spontaneous cell death and as a control for maximum tumor cell death these cells were lysed with 0.1% triton. The ratio of NK cells: tumor cells is 5:1. n=5. P values were calculated using a RM one-way ANOVA followed by Dunnett’s *post hoc* multiple comparison test for **(A)** and a Friedman one-way ANOVA followed by Dunn’s *post hoc* multiple comparison test for **(C)**.

## Discussion

Immune cells are an important part of the TME and can directly affect tumor control or growth. The exact immune cell composition is highly variable and, among other things, dependent on tumor cell type and stage (14, 38). Lymphocytes can infiltrate tumors and act as potent killers. However, tumors often induce an immunosuppressive microenvironment by recruiting Tregs and MDSCs to prevent these potent killers from restricting tumor growth.

Since the presence of increased numbers of neutrophils - in blood (as neutrophil-lymphocyte ratio) or within the TME itself - are associated with a worse outcome for patients, it is important to dissect and further understand PMN-MDSC activity exerted in the TME. Given that different lymphocyte subsets are present in the TME, we investigated whether neutrophils showed selectivity towards specific lymphocyte subsets. In this study, we showed that several αβ T cell subsets (CD4^+^ and CD8^+^; naive or memory, Tregs) are vulnerable to PMN-MDSC activity, while innate γδ T cells and NK cells are not. This raises the question what explains that all T cell subsets that are suppressed by PMN-MDSC activity have an αβ-TCR. Several mechanisms are necessary for PMN-MDSC activity, including; neutrophil ROS production, β2-mediated cell-cell contact, trogocytosis and degranulation. Because most of these functions are reliant on neutrophils rather than a specific T cell subset or on NK cells, we hypothesize that mainly cell-cell contact determines whether there is PMN-MDSC activity on immune cells or not. The main difference between αβ T cells versus γδ T cells and NK cells is the expression of the αβ TCR and (a relative high expression) of CD3, CD4 and CD8. Meanwhile, activated γδ T cells and NK cells express many innate associated receptors and ligands which may partially inhibit or protect them against MDSC activity. Which proteins, or the absence of them, render αβ-TCR positive T cells more susceptible to neutrophil mediated suppression is yet to be revealed, which hints towards further research into this important observation. One limitation of this study is that using the CD8-memory T cell isolation kit does not lead to pure CD8^+^ memory T cells. Isolating T cells with this antibody microbead cocktail leads to a mix of memory-like T cell subsets, shown by CD27 expression, with unknown effector functions. This is not found for CD4memory T cells. Furthermore, the CD8^+^ memory T cell fraction is slightly contaminated with CD8^+^ terminally differentiated effector memory T cells (TEMRAs), which are CD45RA^+^/CD27^-^ (as shown in **Supplementary Figure 3B**) (39). Therefore, isolating CD45RA and CD45RO positive CD8^+^ T cells and comparing them to each other does not give a complete picture. Nevertheless, we still observe the same trend as for the CD4^+^ naive versus memory T cells, both showing vulnerability towards PMN-MDSC activity.

Another limitation of this study is that Tregs and NK cells were first cultured before starting the MDSC assay, while the other lymphocyte populations were not. For Tregs this is needed to obtain enough cells for starting a co-culture with neutrophils while for NK cells this was mainly needed as recovery time before starting to proliferate. Since we do observe PMN-MDSC activity towards Tregs but not towards NK cells, we believe that prior culturing of the lymphocyte populations does not influence PMN-MDSC activity. Furthermore, this was tested for NK cells, where both cells that were first cultured or immediately used in a co-culture with neutrophils were not vulnerable towards PMN-MDSC activity. Previous research showed inconclusive results regarding PMN-MDSC activity and the effect on γδ T cells (21, 22). One study showed no effect on γδ T cell proliferation while another study did show γδ T cell suppression with neutrophils. We found that there is not effect of neutrophil suppression on γδ T cells, which aligns with the first study. The discrepancy found could partially be explained by different γδ T cell stimulation strategies including phosphoantigens bromohydrin pyrophosphate (IPH11) and (E)-1-hydroxy-2-methylbut-2-enyl 4-diphosphate (HMBPP). Moreover, this suppressive effect of neutrophils on γδ T cells, was already observed without prior pre-activation of the neutrophils. Although nothing has been reported regarding IPH11 stimulation to neutrophils, HMBPP has been reported to induce neutrophil activation (40).

Regarding NK cells, inhibition of NK cell-mediated tumor cell cytotoxicity by PMN-MDSC activity has been previously reported (23). However, instead of neutrophils from healthy donors receiving G-CSF for stem cell mobilization, we used healthy donor neutrophils and stimulated them with TNFα to induce PMN-MDSC activity, based on our previous observation that neutrophils do not show immunosuppressive activity without prior activation (19).

For Tregs, adding either TNFα or neutrophils did increase cell proliferation. As known from literature, Tregs express high levels of TNF receptor 2 (TNFR2) and TNFR2 ligation in Tregs can enhance IL-2 induced T cell proliferation (41–44). Conventional T cells do not respond to TNFα (45). In the context of inflammation, this could mean that presence of TNFα stimulates Tregs to become active and thereby play a suppressive role in the inflammatory environment. However, the presence of neutrophils could counteract this suppression.

Since γδ T cells and NK cells are not vulnerable to PMN-MDSC activity *in vitro*, the presence of suppressive neutrophils in the TME might be inconsequential for the anti-tumor effector function of these cells. However, high numbers of Tregs could still play an unfavorable suppressive role with respect to these cytotoxic innate lymphocytes. The presence of neutrophils could counteract this suppressive effect of Tregs by suppressing these cells. While the presence of neutrophils in the TME in general is correlated with a worse patient outcome due to its immunosuppressive activity, though our data gives the story another possible layer of complexity depending on immune cell infiltration. Our data suggest that subset specific protein expression (or production) may determine the (level of) susceptibility to PMN-MDSC activity. Moreover, local immune cell proportions, their intratumoral localization and anti-tumor cytotoxic contribution might play an important, but yet not fully clarified role, being tumor cell type specific and even patient specific. Investigating what causes differences in vulnerability towards PMN-MDSC activity between T cells and NK cells will give more insight into the process itself, potentially leading to αβ engineering CAR-T cells equipped to resist immunosuppressive activity of neutrophils. Even more, it could contribute to target PMN-MDSC activity in cancer patients as such, by defining the most critical interactions that are exploited by neutrophils to suppress αβ T cells (19). Patients with tumors known to have high neutrophil infiltration may benefit from antibody-based immunotherapies that block these interactions. On the other side, focusing on innate γδ T cells and NK cells as cellular products that are not vulnerable for PMN-MDSC activity could have important advantages. By pinpointing the proteins that protect anti-tumor effector cells from suppression by neutrophils may help shape therapies to improve tumor infiltration by lymphocytes, their survival and intra tumoral efficacy. In conclusion, our data underlines that neutrophils behave differently towards most αβ T cells by suppressing them, while the innate γδ T cells and NK cells are not affected, opening up possible intervention strategies for immunotherapy.

## Data Availability

The raw data supporting the conclusions of this article will be made available by the authors, without undue reservation.

## Supplementary material

Supplementary Figure 1 Purity of isolated CD4^+^ and CD8^+^ T cells and analysis of damaged T cells over time. **(A)** Purity of CD4^+^ and CD8^+^ T cells measured by flow cytometry using a mixture of anti-CD4-APC and anti-CD8-PerCP-Cy5.5 antibodies, directly after isolation. **(B)** T cells with and without neutrophils were stimulated with aCD3/28 + TNFα. Analysis of damaged T cells was performed from day 0 to day 4 by comparing FSC/SSC. n=2.

Supplementary Figure 2 Purity of isolated CD4^+^ naive and memory T cells.

Supplementary Figure 3 Purity of isolated CD8^+^ naive and memory T cells. **(A)** Purity of CD8naive and CD8memory T cells measured by flow cytometry using a mixture of anti-CD8-PerCP-Cy5.5, anti-CD45RO-PB and anti-CD45RA-PECy7 antibodies, directly after isolation. **(B)** Purity of CD8naive and CD8memory T cells measured by flow cytometry using a mixture of anti-CD45RA-PECy7 and anti-CD27-FITC antibodies, directly after isolation.

Supplementary Figure 4 CD8naive and memory T cells are both vulnerable to PMN-MDSC activity. **(A)** CD8naive and CD8memory T cell proliferation. n=3. **(B)** CD8naive and CD8memory damaged T cells resulting from a coculture with neutrophils for four days. n=3. P values were calculated using a RM one-way ANOVA followed by Dunnett’s *post hoc* multiple comparison test.

Supplementary Figure 5 Purity of isolated cells. **(A)** Purity of Tregs measured by flow cytometry one week after isolation by using an intracellular staining with anti-FOXP3-PECy7 and anti-Helios-PerCP-Cy5.5. **(B)** Purity of γδ T cells measured by flow cytometry directly after isolation, determined with anti-γδ-TCR-PE. **(C)** Purity of NK cells measured by flow cytometry one week after isolation by a mixture of anti-CD56-PerCPCy5.5 and anti-CD3-PB.

Supplementary Video 1 Trogocytosis of CD4^+^ (magenta) and CD8^+^ (green) T cells by neutrophils (orange) in presence of aCD3/28 and TNFα as imaged by confocal microscopy.
